# Radiotherapy combined with immune checkpoint inhibitors in locally advanced/metastatic esophageal squamous cell carcinoma: clinical trials, efficacy and future directions

**DOI:** 10.3389/fimmu.2023.1177085

**Published:** 2023-05-30

**Authors:** Mengjie Jiang, Yujie Hu, Gang Lin, Chao Chen, Huafeng Li

**Affiliations:** Department of Radiotherapy, The First Affiliated Hospital of Zhejiang Chinese Medical University (Zhejiang Provincial Hospital of Chinese Medicine), Hangzhou, China

**Keywords:** esophageal squamous cell carcinoma (ESCC), radiotherapy, immune checkpoint inhibitor (ICI), combination efficacy, adverse event (AE)

## Abstract

Esophageal squamous cell carcinoma (ESCC) is a common malignancy worldwide and often diagnosed at advanced stages with poor prognosis. Combination of radiotherapy and immunotherapy seems to be a promising approach for treating ESCC. This comprehensive review article summarizes the current state of combination of radiotherapy and immunotherapy in locally advanced/metastatic ESCC, delineates the clinical trials that merit attention, and outlines unresolved issues and future research directions in this field. The clinical trial findings suggest that radio-immunotherapy combination may improve tumor response and overall survival with manageable side effects, highlighting the importance of patient selection and the necessity for further research to optimize treatment strategies. Issues such as irradiation dosage, fractionation regimen, irradiation site and technique of radiotherapy, as well as the timing, sequence and duration of combination therapy will all affect treatment outcomes, justifying further in-depth investigation.

## 1 Introduction

Esophageal cancer, a malignancy that poses a significant threat to human health, is ranked seventh in incidence and sixth in mortality worldwide (1). Based on their histological features, they can be divided into esophageal squamous cell carcinoma (ESCC) and esophageal adenocarcinoma (EAC), which differ substantially in pathogenesis, biological behavior, treatment and prognosis. Locally advanced esophageal cancer refers to those with tumor invasion of local structures or regional lymph node metastasis without distant metastasis (ie, American Joint Committee on Cancer stage ≥T2 or N+, M0) (2), accounting for the majority of clinical cases, and can be divided into resectable and unresectable groups based on the feasibility of radical resection.

Although significant progress has been achieved in the treatment of esophageal cancer, its efficacy remains unsatisfactory. Immunotherapy, exemplified by immune checkpoint inhibitors (ICIs), has emerged as a promising new therapeutic strategy that continues to revolutionize the treatment of esophageal cancer. However, the employment of a single-agent ICI approach is constrained by a response rate of only approximately 20% (3–6). To overcome this limitation, researchers have explored the combination of immunotherapy with other treatment modalities. Radiotherapy is widely applied in each stage of esophageal cancer and has garnered considerable attention in combination with immunotherapy, following the KEYNOTE-001 study, which revealed a superior efficacy of ICI in patients who had previously received radiotherapy (7).

For resectable or potentially resectable locally advanced ESCC, the current treatment paradigm established by the CROSS study and the NEOCRTEC5010 study is neoadjuvant chemoradiotherapy followed by surgery, which has achieved a pathologic complete response (pCR) rate of 43-49%, a 5-year overall survival (OS) rate of 60%, and a 10-year OS rate of 46% (8–11). Despite significant advances, neoadjuvant chemoradiotherapy remains a topic of debate, with ongoing discussions on the optimal modality of neoadjuvant therapy. Could the addition of immunotherapy to neoadjuvant chemoradiotherapy further improve the outcomes? Alternatively, could neoadjuvant chemoradiotherapy be replaced by neoadjuvant chemotherapy or immunotherapy? Within the framework of trimodality therapy (radiotherapy, chemotherapy and surgery), distant metastasis remains the most predominant mode of subsequent failure (12), and minimal residual disease (MRD) may be the source of relapse and metastasis; could it be eradicated by intensified postoperative adjuvant immunotherapy? For unresectable locally advanced esophageal cancer, definitive chemoradiotherapy is the current standard treatment. Despite multiple studies have explored the optimal chemotherapy regimens and alternative radiotherapy dose fractionation, the complete response rate remains low and the local recurrence rate remains high, with a 3-year OS rate of 40-55% and a 5-year OS rate of only 20-25% (13–17). Might the integration of immunotherapy, administered concurrently with or subsequent to definitive chemoradiotherapy, emulate the favorable outcomes of the PACIFIC protocol in lung cancer? Metastatic esophageal cancer is currently treated with ICI combined with chemotherapy as the standard first-line treatment (18, 19), but the efficiency has attained a plateau. Empirical evidence indicates that supplementing well-managed systemic therapy with aggressive local radiotherapy can not only enhance the nutritional status and improve the quality of life but also stimulate the systemic anti-tumor immune response, leading to substantial survival advantages (20–23). The relatively high proportion of oligometastasis in ESCC patients highlights the importance of exploring the combination of radiotherapy and systemic therapy as an urgent research topic. Resolutions to these inquiries can only be attained through ongoing preclinical and clinical investigations.

In China, squamous cell carcinoma is the predominant histological subtype of esophageal cancer, accounting for more than half of the global morbidity and mortality (1). Despite the abundance of publications reporting the synergistic effects and underlying mechanisms of radiotherapy and immunotherapy (24, 25), research on radio-immunotherapy combinations remains in its infancy and is confronted with various challenges, including several controversial issues that need to be resolved before its potential widespread clinical application. In this article, we present a comprehensive review of the application of radiotherapy in combination with ICIs in patients with locally advanced/metastatic ESCC, delineate the clinical trials that merit particular attention, and synthesize some of the unresolved issues and future research directions in this field.

## 2 Synergistic mechanisms of radiotherapy combined with ICIs

Radiotherapy is not only efficacious in directly eradicating tumor cells, but also intricately associated with the local immune microenvironment and systemic immune status. In recent years, there have been more and more basic research and clinical trials focusing on the combination of radiotherapy with ICIs. A multitude of studies has demonstrated that the combination can enhance the abscopal effect and antitumor immune memory, leading to a favorable therapeutic outcome (26, 27). The synergistic mechanism of radiotherapy combined with immunotherapy can be summarized as follows (28, 29): Radiation causes cancer cells to undergo immunogenic cell death, releases tumor-associated antigens, which are recognized by antigen-presenting cells and presented to T cells, generating an effect similar to “*in situ* vaccines” and activating systemic adaptive immune responses to eliminate tumors. Furthermore, radiotherapy reprograms the tumor microenvironment by upregulating the expression of PD-L1 in the tumor microenvironment, regulating various immune cells through cytokines and chemokines, resulting in the conversion of immunologically “cold” tumors into “hot” tumors, making them more amenable to immunotherapy (30, 31). Reciprocally, ICIs not only activate cytotoxic T cells to attack tumor cells, but also normalize tumor vasculature, enhance tissue perfusion to mitigate tumor hypoxia and increase sensitivity to radiotherapy (32, 33).

## 3 Clinical trials testing the combination of radiotherapy with ICI in ESCC

Systematic searches were performed in PubMed, Google Scholar, and ClinicalTrial.gov databases up to February 2023 to gather information on clinical trials that explore the efficacy of the combination of radiotherapy and ICIs for the treatment of ESCC. Our findings have been collated in **Table 1**.

**Table 1:** Clinical trials investigating the combination of radiotherapy and immune checkpoint inhibitors in esophageal squamous cell carcinoma.

Identifier	Phase	Participants	Radiotherapy Regimen	Immunotherapy Regimen	Chemotherapy Regimen	Primary Outcome	Secondary Outcome	Published Results	Author	Time
Neoadjuvant therapy										
NCT02844075	II	28	44.1GY/21F	Neoadjuvant period: Pembrolizumab 200mg Q3W *2 Postoperative period: Pembrolizumab 200mg Q2W (maximum 2 years)	paclitaxel+carboplatin	pCR	DFS OS AE	pCR 46.1%, 12m-OS 80.8%, 18m-OS 73.1%	Lee S (34)	2019
NCT03792347 PALACE-1	Ib	20	41.4GY/23F	Pembrolizumab 2 mg/kg Q3W *2	paclitaxel+carboplatin	AE	Feasibility pCR	Grade III AE 65%, Grade V AE 1, pCR 55.6%	Li C (35)	2021
NCT04435197 PALACE-2	II	143	41.4GY/23F	Pembrolizumab 200mg Q3W *2	albumin paclitaxel+carboplatin	pCR	DFS OS			
NCT04929392	II	24	41.4GY/23F	Pembrolizumab Q3W *2	paclitaxel+carboplatin; lenvatinib	pCR/cCR	AE DFS OS			
NCT05541445	Ib/II	40	44GY/22F	Neoadjuvant period: Induction Pembrolizumab+chemotherapy 200mg Q3W *2, Sequential Pembrolizumab+chemoradiotherapy 200mg Q3W *2; Postoperative period: Pembrolizumab 200mg Q3W (maximum 1 year)	albumin paclitaxel+ cisplatin	MPR	R0 rate DFS LRRFS OS			
NCT03544736 INEC	I/II	30	Palliative: 20-50GY(2-4GY/F) Definitive: 50.4GY/28F Neoadjuvant: 41.4GY/23F	Palliative: Nivolumab 240mg Q2W/360mg Q3W/480mg Q4W (maximum 2 years); Definitive: Nivolumab 240mg Q2W during radiotherapy, 480mg Q4W (maximum 1 year); Neoadjuvant: Nivolumab 240mg Q2W; Postoperative: Nivolumab 480mg Q4W (maximum 1 year)	paclitaxel+carboplatin	AE	ORR PFS OS QoL			
NCT04229459^a^	II	31	50.4GY/28F	Nivolumab 3mg/kg Q2W *3	cisplatin+5-FU; cetuximab	pCR PFS AE	OS			
NCT03044613	Ib	32	standard care dose	Induction Nivolumab 240mg ± Relatlimab 80mg Q2W *2 Concurrent Nivolumab 240mg ± Relatlimab 80mg Q2W *3	paclitaxel+carboplatin	AE	feasibility pCR OS RFS			
ChCTR2100045104 SCALE-1	Ib	20	30GY/12F	Toripalimab 240mg Q3W *2	paclitaxel+carboplatin	AE	pCR MPR	pCR 55%, MPR 80%	Jiang N (36)	2022
NCT05424432 SCALE-2	II	63	30GY/12F	Toripalimab 240mg Q3W *2	paclitaxel+carboplatin	pCR	DFS OS AE QoL			
​NCT04006041	II	44	44GY/20F	Toripalimab 240mg Q3W *2	paclitaxel+cisplatin	pCR	OS DFS AE R0 rate			
NCT04177875	II	44	40Gy/20F	Toripalimab 240mg Q3W *2	docetaxel/albumin paclitaxel+cisplatin	MPR ORR	DFS OS AE			
NCT04437212^a^	II	20	41.4GY/23F	Neoadjuvant period: Toripalimab 240 mg Q3W *2 Postoperative period: Toripalimab 240 mg Q3W *4	paclitaxel+cisplatin	MPR	DFS OS AE	pCR 54%, MPR 77%, Grade 3-4 AE 54%	Xu X (37)	2022
​NCT04644250	II	32	41.4GY/23F	Toripalimab 240mg Q3W *3	paclitaxel liposome+carboplatin	pCR	AE OS DFS MPR ORR R0 rate			
NCT04888403	II	45	41.4GY/23F	Induction Toripalimab 240mg *1, concurrent Toripalimab 240mg Q3W *4	albumin paclitaxel+nedaplatin	pCR	MPR DFS R0 rate			
NCT02735239 LUD2015-005	I/II	73	standard care dose	Durvalumab 750mg Q2W	paclitaxel+carboplatin	AE	ORR PFS OS			
NCT04568200	II	60	41.4GY/23F	Durvalumab 1500mg Q3W *4	paclitaxel+carboplatin	ORR	feasibility AE R0 rate PFS OS			
NCT04776590^a^CRISEC	II	30	41.4GY/23F	Tislelizumab 200mg Q3W *3	albumin paclitaxel+carboplatin	pCR	DFS OS	pCR 46.7%, MPR 86.7%	Yang J (38)	2022
NCT04974047	II	70	40GY/20F	Tislelizumab 200mg Q3W *3	paclitaxel + cisplatin; cisplatin+5-FU	pCR	R0 Rate DFS EFS ORR AE			
NCT05189730^a^ ETNT	II	80	40GY/20F	Neoadjuvant period: Tislelizumab 200mg Q3W *2; Maintenance period: Tislelizumab 200mg Q3W (maximum 1 year)	paclitaxel+carboplatin	pCR AE	cCR MPR ORR R0 rate EFS OS			
NCT04973306 iCROSS	II/III	176	41.4GY/23F	Tislelizumab 200mg Q3W *2	paclitaxel+carboplatin	pCR OS	AE PFS R0 rate RFS			
NCT03165994	II	34	50.4GY/28F	Sotigalimab 0.3mg/kg Q3W *3	paclitaxel+carboplatin	pCR	R0 rate AE ORR	pCR 36% (7/23 adenocarcinomas, 3/5 squamous cell), MPR 64%	Ko AH (39)	2022
NCT03490292^a^	I/II	22	41.4GY/23F	Neoadjuvant period: Avelumab 10 mg/kg Q2W *3 Postoperative period: Avelumab 10 mg/kg Q2W *6	paclitaxel+carboplatin	pCR	AE DFS R0 rate	pCR 26% (3/16 adenocarcinomas, 2/3 squamous cell), MPR 42%	Uboha NV (40)	2022
NCT05650216 NICE-RT	II	50	Primary lesion and adjacent lymph nodes: 41.4GY/23F; Abscopal lymph node 2GY/4F	Neoadjuvant period: Camrelizumab 200mg Q3W *2; Postoperative period: Camrelizumab 200mg Q3W (maximum 1 year)	albumin paclitaxel+carboplatin	AE pCR	MPR ORR EFS OS			
NCT05355168 NCRCN	I/II	57	41.4 GY/23F	Camrelizumab	paclitaxel+carboplatin; nimotuzumab	pCR MPR	AE DFS			
NCT05507411 WATCHER	II	100	41.4GY/23F	Camrelizumab 200mg Q3W	albumin paclitaxel+carboplatin	DFS	OS EFS cCR pCR MPR			
NCT05043688 NICE-2	II	204	41.4GY/23F	Neoadjuvant period: Camrelizumab 200mg Q3W *2 Postoperative period: Camrelizumab 200mg Q3W (maximum 1 year)	albumin paclitaxel/paclitaxel +carboplatin	pCR	OS R0 rate EFS DFS AE			
NCT05176002	I/II	26	41.4GY/23F	Camrelizumab	NA	MPR AE	pCR			
NCT03940001	I	20	41.4GY/23F	Sintilimab 200mg Q3W	paclitaxel+carboplatin	AE pCR MPR	NA			
NCT05357846	III	422	40 or 45GY/20F	Sintilimab 200mg Q3W *2	paclitaxel+cisplatin	OS	PFS pCR R0 rate AE			
Adjuvant therapy^b^										
NCT02520453	II	86	neoadjuvant 44GY/22F	Durvalumab 20 mg/Kg Q4W (maximum 1 year)	neoadjuvant cisplatin+5-FU	DFS	OS	No difference in DFS or OS between Durvalumab and placebo	Park S (41)	2022
NCT02743494 CheckMate 577	III	794	neoadjuvant 41.4–50.4GY	Nivolumab 240 mg Q2W *8 followed by 480 mg Q4W *9	neoadjuvant paclitaxel+carboplatin; cisplatin+5-FU	DFS	OS ORR	DFS: Nivolumab 22.4m vs placebo 11.0m; Grade 3-4 AE: Nivolumab 13% vs placebo 6%	Kelly RJ (42)	2021
NCT04741490	NA	20	adjuvant 45-55GY (1.8-2.0GY/F)	Camrelizumab 200mg Q3W *6	NA	DFS	PFS OS			
NCT03322267	II	26	adjuvant 18-26GY/10-13F	Pembrolizumab 200mg Q3W *18	cisplatin	RFS	OS AE			
NCT05103501 SINCERE	II	54	NA	Pembrolizumab 200mg Q3W (maximum 2 years)	cisplatin+5-FU	DFS	OS			
Definitive therapy										
NCT03222440	Ib	20	54-60GY/30F	Camrelizumab 200mg Q2W *16	NA	AE	ORR PFS OS	ORR 74%, mOS 16.7m, mPFS 11.7m	Zhang W (43)	2021
NCT03671265	Ib	20	60GY/30F	Camrelizumab 200mg Q2W *16	docetaxel+cisplatin; apatinib	AE	ORR PFS OS	ORR 65%, 24m-OS 69.6%, 24m-PFS 65.0%	Zhang W (44)	2021
NCT04426955 ESCORT-CRT	III	396	50.4GY/28F	Camrelizumab 200mg Q3W	paclitaxel+cisplatin	PFS	OS ORR AE			
NCT05624099	II	226	50GY/30F	Camrelizumab 200mg Q3W	paclitaxel+platinum	ORR	OS PFS			
NCT04210115 KEYNOTE-975	III	700	50/25F 60GY/30F	Pembrolizumab 200mg Q3W *8 followed by 400mg Q6W *5	cisplatin+5-FU; oxaliplatin+leucovorin+5-FU	EFS OS	AE			
NCT03957590 RATIONALE311	III	370	50.4GY/28F	Tislelizumab 200mg Q3W (maximum 2 years)	paclitaxel+cisplatin	PFS	ORR OS AE QoL			
​NCT05515315^a^	II	93	50-60GY/25-30F	Tislelizumab 200mg Q3W	albumin paclitaxel+nedaplatin	PFS	ORR OS			
NCT05520619​ EC-CRT-002	II	114	50.4GY/28F	Tislelizumab 200mg Q3W *4 ± maintenance Tislelizumab 200mg Q3W *12	paclitaxel+cisplatin	PFS	OS cCR AE			
NCT03377400	II	40	60.2/64.5GY	Durvalumab+Tremelimumab Q3W *4, Durvalumab Q4W (maximum 2 years)	cisplatin+5-FU	PFS	OS	24m-PFS 57.5%, 24m-OS 75%	Park S (45)	2022
NCT04550260 KUNLUN	III	600	50-64GY	Durvalumab (maximum 2 years)	cisplatin+5-FU; cisplatin+capecitabine	PFS	OS AE			
NCT03777813 ARION	II	120	Macroscopic disease: 50GY/25F; Adjacent peri tumoral mucosis and prophylactic lymph node: 45GY/25F	Durvalumab 1500mg Q4W (maximum 1 year)	oxaliplatin+leucovorin+5-FU	PFS	OS AE QoL			
NCT04851132	II	33	59.92GY/28F	Durvalumab 1000mg Q3W *18	NA	PFS	OS ORR DCR AE QoL			
NCT03437200 CRUCIAL	II	130	50GY/25F	Nivolumab 240mg Q2W (maximum 1 year); Ipilimumab 1 mg/kg Q6W (maximum 1 year)	oxaliplatin+leucovorin+5-FU	PFS	ORR OS			
NCT03278626	I/II	44	50.4GY/28F	Nivolumab 240mg Q2W	paclitaxel+carboplatin	AE cCR	PFS OS			
NCT04005170 EC-CRT-001	II	42	50.4GY/28F	Toripalimab 240mg Q3W (maximum 1 year)	paclitaxel+cisplatin	cCR	OS PFS AE	cCR 62%, 1y-OS 78.4%,1y-PFS 54.5%	Zhu Y (46)	2023
NCT04844385^a^	II	83	60GY/24F	Toripalimab 240mg Q3W *2	albumin paclitaxel+nedaplatin;capecitabine	PFS	OS cCR AE QoL			
NCT04602013	II	53	60-66GY/30-33F	Sintilimab 200mg Q3W	albumin paclitaxel+cisplatin	PFS	ORR OS QoL			
NCT05621707^a^ RICE	II	50	NR	Sintilimab 200mg Q3W (maximum 1 year)	albumin paclitaxel+ carboplatin	OS	PFS AE			
NCT04821778	III	2000	50-66GY/25-30F	Anti-PD-1/PD-L1 Antibody	paclitaxel/platinum/5-FU	OS	PFS AE LRFS DMFS			
Consolidative therapy										
UMIN000034373 TENERGY EPOC1802	II	50	definitive 60GY/30F	Atezolizumab 1200mg Q3W (maximum 1 year)	cisplatin+5-FU	cCR	PFS OS ORR AE	cCR 42.1%, mPFS 3.2m, mOS 31.0m	Bando H (47)	2022
NCT04543617 SKYSCRAPER-07	III	750	standard care dose	Atezolizumab 1200mg Q3W *17; Tiragolumab 600mg Q3W *17	platinum-based	PFS OS	ORR DOR AE			
NCT04286958	II	40	definitive 50-60GY	Camrelizumab 200 mg Q2W (maximum 1 year)		PFS	ORR DOR OS AE	ORR 91.7%	Wang J (48)	2022
NCT03817658	II	725	45-50GY (1.8-2GY/F)	Camrelizumab 200 mg, or 3 mg/kg for weight <50 kg Q2W (maximum 1 year)	cisplatin+capecitabine	PFS OS	ORR DOR QoL AE			
NCT04514835	II	44	50-50.4GY/25-28F	Sintilimab 200mg Q3W (maximum 1 year)	cisplatin+capecitabine	PFS	ORR OS			
NCT04212598	II	40	50.4GY/28F, patients with residual disease boost to 61.2 Gy/34F	Sintilimab 200mg Q3W (maximum 1 year)	NR	PFS	OS AE			
NCT04054518 DESC	II	22	definitive ≥ 50GY	Durvalumab 1500mg Q4W (maximum 1 year)	definitive platinum-based	PFS	AE OS			
Palliative therapy										
ChiCTR2100046715^a^ TR-EAT	II	30	30–50GY/15–25F, SBRT for oligometasis 4-8GY/F for 3-5F	Toripalimab 240mg Q3W (maximum 1 year)	paclitaxel+carboplatin	PFS	ORR DCR DOR OS AE QoL	ORR 81.8% DCR 100%	Wu L (49)	2022
NCT02830594	II	14	palliative radiotherapy	Pembrolizumab Q3W (maximum 2 years)	NA	Changes in Non-irradiated Sites	AE ORR PFS OS			
NCT05628610	II	130	50GY/30F	Tislelizumab 200mg Q3W	paclitaxel+platinum	ORR	PFS			
NCT04821765	II	35	50-60GY (1.8-2GY/F or 3-4GY/F)	Tislelizumab 200mg Q3W (maximum 1 year)	albumin paclitaxel+cisplatin	LCR	AE PFS OS ORR			
NCT05547828	II	20	40GY/20F (primary tumor and metastases)	Tislelizumab 200mg Q3W	albumin paclitaxel	ORR	DCR PFS OS			
ChiCTR2000040533	II	49	primary tumor(40GY/20F), metastases(30GY/10F)	Camrelizumab 200mg Q3W	irinotecan	PFS	OS ORR DCR CBR safety ARR ACR	ORR 40.8% DCR 75.5% mPFS 6.9m, mOS 12.8m	Zhao W (50)	2023
NCT04390945^c^	II	62	50-50.4GY/25-28F	Camrelizumab 200mg Q2W	capecitabine	PFS	OS ORR			
NCT04404491^c^	III	240	50-50.4GY/25-28F	Camrelizumab 200mg Q2W *5	oxaliplatin+capecitabine	AE PFS	ORR OS			
NCT04512417^a^	II	63	SBRT, 8GY/F 3-5F; conventional ≥30GY	Camrelizumab 200mg Q3W (maximum 2 years)	NA	PFS	ORR OS AE			
NCT05183958^a^	II	118	SBRT, 8GY/F 3-5F; Conventional ≥40GY	Camrelizumab 200mg Q3W (maximum 2 years)	paclitaxel+platinum; cisplatin+5-FU; capecitabine+cisplatin	PFS	ORR OS AE			
ACTRN12619001371189 PALEO	II	54	hypofractionated (30GY/10F) to primary lesion in week 1 + SBRT (24GY/3F) to metastasis in week 7	Durvalumab 1500mg Q4W (maximum 2 years)	paclitaxel+carboplatin	PFS	QoL ORR AE PFS OS			
ChiCTR1900027161^a c^	II	27	NR	Sintilimab 200mg Q3W (maximum 1 year)	NR	PFS	ORR DCR OS	mPFS 8.3m mOS 15.7m ORR 33.3% DCR 95.2%	Li B (51)	2022
NCT05512520^a^ EC-CRT-003	II	126	45-50.4GY/25-28F	anti-PD1 Q3W	fluoropyrimidine or taxane-based platinum doublet; capecitabine	PFS	OS ORR AE			

pCR, pathologic complete response; DFS, disease-free survival; OS, overall survival; AE, adverse event; cCR, clinical complete response; MPR, major pathologic response; R0 rate, R0 resection rate; LRRFS, local-regional recurrence free survival; ORR, objective response rate; PFS, progression-free survival; QoL, quality of life; 5-FU, 5-fluorouracil; RFS, recurrence-free survival; EFS, event-free survival; DCR, disease control rate; LRFS, locoregional recurrence free survival; NR: not reported; DMFS, distant metastasis free survival; SBRT, stereotactic body radiation therapy; DOR, duration of objective response; LCR, locoregional control rate; NA, not applicable.

a, radiotherapy and immunotherapy administered unsimultaneously;

b, no specific neoadjuvant chemoradiotherapy regimen was required for enrollment, we only listed the recommended or highest percentage regimens;

c, recruit locally recurrent esophageal squamous cell carcinoma without distant metastasis.

The colored texts in revised manuscript represent the revised portions, and the colored numbers represent the reference.

### 3.1 Locally advanced resectable/potentially resectable ESCC

#### 3.1.1 Preoperative neoadjuvant therapy

The Korean phase II trial of neoadjuvant chemoradiotherapy combined with Pembrolizumab for ESCC showed a pCR rate of 46.1% (34), which is comparable to the squamous cell subgroup in the CROSS study, as well as in the NEOCRTEC5010 study (8, 10). Among 28 enrolled patients, two died before surgery due to hematemesis and another two died after surgery due to acute lung injury (34). Although the statistics indicate no statistical difference in surgical risk or postoperative complications when compared to patients who underwent conventional neoadjuvant chemoradiotherapy during the same hospitalization period, the adverse effects of the combination should not be overlooked (52). In 2021, Chinese scholars reported results from the similarly designed PALACE-1 trial, which enrolled 20 patients and achieved a promising pCR rate of 55.6% and a notable major pathologic response (MPR) rate of 89%. However, 65% of patients experienced grade 3 or higher adverse events (AEs), and one patient died due to toxicity (35). The subsequent multicenter PALACE-2 study is currently recruiting to further investigate the efficacy and safety of neoadjuvant chemoradiotherapy combined with Pembrolizumab in resectable ESCC (53). In the phase Ib SCALE-1 trial, short-course neoadjuvant chemoradiotherapy (30GY/12F) plus anti-PD-1 Toripalimab followed by esophagectomy achieved an impressive pCR rate of 55% and MPR rate of 80%. Severe treatment-related consisted primarily of myelosuppression (leukopenia and neutropenia) and gastrointestinal toxicity (anorexia and nausea). Grade 3 perioperative complications occurred in 3 of the 20 patients who underwent surgery (36). A novel CD40 agonistic monoclonal antibody, Sotigalimab, in combination with chemoradiotherapy, has shown a pCR rate of up to 60% in squamous cell carcinoma. However, the sample size is too limited, and further studies involving larger sample sizes are required to validate the findings (39).

In consideration of safety concerns, several studies have investigated the sequential administration of ICIs and radiotherapy rather than simultaneous treatment. The interim results of phase II clinical trial of neoadjuvant chemoradiotherapy followed by sequential Tislelizumab showed that among 15 patients undergoing radical surgery, the pCR rate was 46.7% and the MPR rate was 86.7%. During neoadjuvant therapy, no grade 3 or higher AEs were reported, and the grade 3 postoperative complication rate was 20.0% (38). In the neoadjuvant chemoradiotherapy plus sequential perioperative Toripalimab study, the pCR rate was 54%, MPR rate was 77%, and grade 3-4 treatment-related AEs occurred in 54% patients (37). Although long-term survival outcomes are not yet available, it has been suggested that paradigm of neoadjuvant chemoradiotherapy plus sequential ICIs may reduce the incidence and severity of AEs.

To further enhance the efficacy of treatment for high-risk patients and avoid overtreatment of low-risk patients, precise individualized multi-modality treatment is under exploration. Participants with a decrease in positron emission tomography (PET) Standardized Uptake Value (SUV)max < 35% will receive Tislelizumab plus chemoradiotherapy as neoadjuvant treatment, while those with a decrease ≥35% will receive Tislelizumab plus chemotherapy without radiotherapy (54). Alternatively, non-clinical complete responders after neoadjuvant chemoradiotherapy will receive additional neoadjuvant immunochemotherapy (55).

In summary, the addition of ICI to neoadjuvant chemoradiotherapy did increase the pCR rate, but also led to increased toxicity, albeit under manageable. Whether the slightly improved pCR and objective remission rates (ORR) could ultimately translate into overall survival benefit still requires long-term follow-up. Two phase III multicenter randomized trials providing insights into the relative benefits of neoadjuvant chemoradiotherapy combined with or without ICI on response rate, overall survival, and safety, is expected to provide more information on this question (56, 57).

#### 3.1.2 Postoperative adjuvant therapy

In the context of adjuvant therapy for resectable ESCC, the randomized, double-blind, placebo-controlled phase III CheckMate 577 study demonstrated that 1-year adjuvant Nivolumab significantly prolonged disease-free survival (DFS) (22.4m vs 11.0 m) and OS (29.7m vs 11.0m) in postoperative non-pCR ESCC patients who underwent radical surgery after standard neoadjuvant chemoradiotherapy (42). However, some scholars have questioned the apparently shorter DFS time observed in the placebo group of CheckMate 577. A retrospective study in the Netherlands collected esophageal patients meeting the inclusion criteria of CheckMate 577 but not receiving adjuvant Nivolumab, revealing a median DFS of 19.7 months, which was much longer than that in placebo population of CheckMate 577 (58). It is also noteworthy that ESCC accounted for only 29% of participants in CheckMate 577, and no DFS benefit was observed in the Asian population or Asia region subgroup (42). In addition, a single-center, randomized, double-blind adjuvant Durvalumab study conducted in Korea yielded negative results, possibly due to the fact that the trial did not exclude pCR patients who were not susceptible to benefit from adjuvant immunotherapy (41).

The CheckMate 577 study represents a significant milestone in the exploration of postoperative adjuvant immunotherapy for esophageal cancer, however, further evidence and investigation are necessary to determine appropriate treatment indications, regimens, and courses. The OS data for CheckMate 577 is not yet available, and the results of additional studies specifically evaluating ICI adjuvant therapy for ESCC are eagerly awaited. The ongoing clinical trial NCT04741490 is evaluating the efficacy of Camrelizumab in combination with radiotherapy in adjuvant phase for ESCC (59). NCT03322267 is enrolling patients who remained at high risk of recurrence (resection margin closure or involvement or ypN+) after neoadjuvant chemoradiotherapy, receiving adjuvant chemoradiotherapy followed by 1-year Pembrolizumab treatment (60). In SINCERE Study, ESCC patients who do not respond to initial neoadjuvant therapy (TRG3 and TRG4) will receive adjuvant chemotherapy combined with 2-years adjuvant pembrolizumab (61).

### 3.2 Locally advanced unresectable ESCC

#### 3.2.1 Definitive chemoradiotherapy combined with ICI

In a clinical study of radiotherapy combined with anti-PD-1 Camrelizumab for locally advanced ESCC, 1 (7.1%) and 13 (92.9%) patients achieved CR and PR, respectively, with no grade 3/4 AEs (62). In a similar phase Ib study, 20 locally advanced ESCC patients treated with radiotherapy plus Camrelizumab achieved an ORR of 74%, with a median OS and progression-free survival (PFS) of 16.7 and 11.7 months, respectively, and 24-month OS and PFS rates of 31.6% and 35.5%, respectively (43). Furthermore, a phase II clinical trial enrolled patients over 70 years old to receive radiotherapy plus Durvarlumab to explore the safety and efficacy of radical radiotherapy combined with immunotherapy in elderly patients (63).

The combination of radiotherapy and ICI has demonstrated success, resulting in a growing interest in integrating ICI into definitive chemoradiotherapy to further improve prognosis. Concurrent administration of Camrelizumab with chemoradiotherapy has shown promising results, with 85.0% and 69.6% 12-month and 24-month OS rates respectively, and 80.0% and 65.0% 12-month and 24-month PFS rates respectively, albeit with 40% of patients experiencing serious treatment-related AEs (44). In the EC-CRT-001 study, 62% of patients receiving Toripalimab combined with chemoradiotherapy achieved complete remission, and the 1-year PFS and OS rates were 54.5% and 78.4%, respectively (46). The randomized, double-blinded, placebo-controlled, multicenter phase III ESCORT-CRT study has been launched to compare the efficacy and safety of Camrelizumab or placebo combined with definitive chemoradiotherapy (64). Other large-scale clinical trials are also underway to assess ICIs administered concurrently with definitive chemoradiotherapy, including Keynote-975 (NCT0421011, Pembrolizumab) (65), RATIONALE 311 (NCT03957590, Tislelizumab) (66), KUNLUN (NCT04550260, Durvalumab) (67, 68), ARION (NCT03777813, Durvalumab) (69). In addition to concurrent administration of ICIs and definitive chemoradiotherapy, clinical studies have explored sequential chemoradiotherapy after immunotherapy to achieve survival benefits on a safe basis (70, 71). The EC-CRT-002 trial compared outcomes of Tislelizumab plus definitive chemoradiotherapy followed by Tislelizumab maintenance or not (72).

Dual immunotherapy (Durvalumab and Tremelimumab) with definitive chemoradiotherapy demonstrated a 2-year PFS of 57.5% and OS of 75%, with the lowest in-field failure rate (17.5%) among all articles published to date (45). However, another trial involving Nivolumab ± Ipilimumab plus chemoradiotherapy had to be terminated due to poor accrual (73). Despite this setback, the favorable prognosis of dual immunotherapy significantly surpasses historical data and does not give rise to a significant increase in toxicity, thus justifying further in-depth investigation.

#### 3.2.2 Definitive chemoradiotherapy followed by consolidation ICI

The TENERGY study evaluated the efficacy of 1-year consolidative Atezolizumab following definitive chemoradiotherapy for unresectable locally advanced ESCC. The interim analysis revealed a clinical complete response rate of 42.1%, with a median PFS of 3.2 months and a median OS of 31.0 months. Furthermore, the 12-month PFS and OS rates were 29.6% and 65.8%, respectively (47, 74). Another study evaluating Camrelizumab as consolidation immunotherapy also demonstrated promising efficacy and manageable toxicity. The interim analysis included 12 patients with a median follow-up of 15 months, 11 of whom had stable disease and one patient had progressive disease, with no grade 3 or 4 AEs (48, 75). In the phase III SKYSCRAPER-07 study (76), participants with locally advanced ESCC who had completed radical chemoradiotherapy without progression were randomized to three groups: a single-immunotherapy group (Atezolizumab), a dual-immunotherapy group (Atezolizumab and the TIGIT inhibitor Tiragolumab), and a placebo-controlled group. The primary endpoints were PFS and OS, and data is not yet available. There are two other studies evaluating the efficacy and safety of consolidation Sintilimab after radical chemoradiotherapy that are worthy of attention. Patients will be evaluated for treatment response 6 weeks after the end of radiotherapy. For patients with residual disease, in addition to 1 year of Sintilimab consolidation (77, 78).

### 3.3 Metastatic/recurrent ESCC

#### 3.3.1 Radiotherapy for primary esophageal lesion

For metastatic and recurrent esophageal cancer, immunotherapy combined with chemotherapy is the current first-line standard of care. Additionally, several clinical trials have investigated the potential benefits of adding irradiation to the primary lesion (50, 79–85). These trials typically enrolled patients with oligometastases (no more than 3-5 metastases in less than 2 organs/lymphatic drainage regions) in good physical status to receive radiation regimens using conventional fractionation with 40-60GY (50, 79–85). Although preclinical studies suggest that hypo fractionated radiotherapy with relatively high daily doses may induce stronger immunostimulating signals, it may not be practical for serial organ like the esophagus. Hence, the optimal dosage of palliative radiotherapy for primary esophageal lesions requires further investigation.

In the TR-EAT study, patients with stage IVB ESCC underwent induction Toripalimab plus chemotherapy, followed by sequential chemoradiotherapy (30–50GY/15–25F, target only the primary esophageal foci and metastatic lymph nodes), and 1-year Toripalimab maintenance (86). The preliminary results revealed an ORR of 81.8%, a disease control rate (DCR) of 100%, with no grade 3 or higher AEs (49). The PALEO study developed a protocol integrating Durvarlumab with chemotherapy and two courses of radiotherapy, giving hypo fractionated radiotherapy (30GY/10F) for primary esophageal lesion in week 1-2, and stereotactic body radiation therapy (SBRT, 24GY/3F) for a single metastasis in week 7 (87). Stage IVB ESCC patients who had failed first-line immunochemotherapy received primary tumor radiotherapy (40GY/20F)/metastatic lesions radiotherapy (30GY/10F) combined with Camrelizumab and irinotecan achieved an ORR of 40.8%, a DCR of 75.5%, with median PFS and OS of 6.9 and 12.8 months, respectively (50). For locoregional recurrent ESCC without distant metastasis, several studies have evaluated the efficacy and safety of Camrelizumab combined with concurrent chemoradiotherapy (80, 88). When Sintilimab was used as consolidation therapy after second-line chemoradiotherapy in locoregional recurrent ESCC, the median PFS and OS were 8.3 and 15.7 months, respectively (51).

#### 3.3.2 Radiotherapy for metastatic lesions

Clinical studies evaluating the combination of systemic therapy with metastatic lesion SBRT are scarce, with the ESO-shanghai10 study being the only one with published results, reporting a median PFS of 13.3 months, median OS of 24.6 months, and a 2-year local control rate of 92.1% (89). However, the study design lacked the participation of immunotherapy. The multicenter ESO-shanghai13 trial, which included a larger number of participants, allowed for the incorporation of immunotherapy and served as a basis for studying the synergistic effect of immunotherapy and radiotherapy (90). Studies on the combination of ICI and SBRT typically involve pan-tumor studies. One such study, the multisite SBRT (30-50GY/3-5F, 2-4 metastases) combined with Pembrolizumab in the treatment of metastasis solid tumors showed an ORR of 13.2%, median PFS of 3.1 months, and median OS of 9.6 months (91). The protocol of the phase II PraG study includes hypo fractionated radiotherapy (5-8GY*3F) for metastatic lesions, followed by PD-1 inhibitors administered within one week after the completion of radiotherapy and a subsequent two-week regimen of Granulocyte macrophage-colony stimulating factor (GM-CSF) (92). The majority of the 54 participants were in poor general condition with high tumor burden after multiple lines of treatment, and had an ORR of 16.7%, DCR of 46.3%, and median PFS and median OS of 4 months and 10.5 months, respectively. This is a noteworthy benefit in the refractory population after multiple therapy, and further clinical research of radiotherapy combined with PD-1 inhibitors combined with GM-CSF and IL-2 is being launched (92, 93).

Unlike high-dose radiotherapy, which directly kills tumor cells and may cause immunogenic cell death, low-dose radiotherapy is intended to reprogram tumor immune microenvironment and reverse tumor immune desertification and immunotherapy resistance (94–96). A *post-hoc* analysis revealed that 58% of the lesions treated with low-dose radiotherapy (total dose of 1-20GY) combined ICI achieved a favorable response (97). In the phase I RACIN study, all visible tumor lesions were administered low-dose radiotherapy (0.5 or 1GY per fraction, every two weeks, total dose 6GY or 13GY), in combination with Nivolumab, low-dose cyclophosphamide or Ipilimumab, aspirin or celecoxib (for activation of antigen-presenting cells). In immunotherapy-naïve patients, 37.5% of irradiated lesions shrank, with overall DCR of 87.5% (94). Low-dose radiotherapy, as a less toxic treatment option, may become a new immunomodulatory modality, especially in patients with large-volume tumors or high metastatic burden.

## 4 Discussion: current progress and future direction

### 4.1 Patient selection and predictive markers

Given the heterogeneity of locally advanced ESCC, a single treatment model for “one-size-fits-all” management cannot be relied upon, and individualized treatment is critical. The screening of dominant populations and predictive biomarkers has emerged as a hot topic in immunotherapy. While PD-L1, as well as tumor mutational load (TMB) and microsatellite instability (MSI are presently the most recognized molecular markers, they are not yet considered ideal predictive markers. The expression levels of the markers fluctuate dynamically, and predictive efficacy can be affected by factors such as which sample, assay, and cut-off value are utilized. In the Korean study of adjuvant Durvalumab, it was found that 73% of patients exhibited alterations in PD-L1 tumor proportion score before and after neoadjuvant therapy, and the post-neoadjuvant PD-L1 expression proved to be more effective in predicting the efficacy of adjuvant treatment compared to the pre-neoadjuvant (41). In CheckMate 577, there was no significant difference in the risk of disease recurrence or death between groups with PD-L1 expression ≥1% and <1%. However, a *post-hoc* analysis stratified by a PD-L1 expression (combined positive score >=5) showed that high PD-L1 expression was associated with longer DFS in the Nivolumab group (42). Furthermore, high PD-L1 expression in peripheral circulating tumor-associated cells, tumor microenvironment, and extracellular vesicles has been demonstrated to be more effective in identifying dominant populations than PD-L1 on the surface of tumor cells (98–101). Dynamic monitoring of changes in PD-L1 expression may also be more valuable than static baseline expression. The use of circulating tumor DNA (ctDNA) to assess MRD shows promise in screening suitable populations for postoperative adjuvant therapy and consolidation therapy after definitive chemoradiotherapy (102–105). Various molecular biomarkers have been identified to predict the response to neoadjuvant chemoradiotherapy for esophageal cancer, as well as various lymphocyte subsets and lymphocyte ratios in the peripheral blood/tumor area, dendritic cells, cytokines, and gut microbiota, have also been reported to have predictive value (35, 44, 106–110). However, these biomarkers are not yet widely utilized in clinical practice, and further research is required to determine whether they can predict the effectiveness of immunotherapy in combination with radiotherapy.

Functional imaging modalities, including pre-, mid-, and post-treatment PET-CT and MRI, offer great potential for providing clues to predict treatment response or prognosis. However, optimization of specific technology and parameters is essential to improve the accuracy of predictions (111–115). Several clinical trials have been conducted to guide treatment decisions based on imaging response (54, 78, 116–118), and there is even novel PET imaging technique that targets tumor PD-L1, which can achieve *in vivo*, noninvasive, and real-time monitoring (119–122).

Advanced ESCC exhibits significant heterogeneity with varying degrees of organ metastases, regional or non-regional lymph node metastases, and therefore the value of radiotherapy application varies. Several large retrospective studies have confirmed that the addition of aggressive primary tumor radiotherapy to the treatment of advanced esophageal cancer has positive implications for patients, but further research is needed on patient selection (22, 123). In the real-world study, the addition of radiotherapy to immunochemotherapy in locally recurrent ESCC significantly prolonged OS (median OS 19.5 vs 7.7 months), while no difference in PFS or OS was observed with or without radiotherapy in the entire cohort (124). This underscores the importance of patient selection. Oligometastatic, as well as limited regional lymph node recurrence in advanced esophageal cancer, are known to have a better prognosis, and aggressive local therapy combined with systemic therapy may offer patients the opportunity for radical treatment and may be a consideration in patient selection. The ESO-shanghai10 trial enrolled highly selected oligometastatic patients with controlled primary sites (≤3 metastases, limited to ≤2 organs), and most patients had only one oligorecurrent and oligometastatic lesion, which may be associated with the impressive outcomes (89). The EC-CRT-003 study included non-regional lymph node metastasis only as a stratification factor that may suggest prognosis and guide treatment (85). Decision tree models have also been used to divide oligometastatic ESCC into different risk groups and screen high-risk patients for early intensive treatment, such as immunochemoradiotherapy (125).

### 4.2 Radiotherapy aspects in combination treatment

Radiotherapy-induced immune response varies widely with the irradiation dose, fractionation regimen, and irradiation site. Therefore, it is essential to evaluate the safety, efficacy, and optimal timing of combined therapy in the context of utilizing radiotherapy in conjunction with ICI for the treatment of ESCC.

#### 4.2.1 Timing of radiotherapy combined with ICI

In general, there are three modes of integrating radiotherapy and ICI: concurrent radiotherapy with ICI, consolidation of ICI following radiotherapy, and radiotherapy subsequent to induction ICI. While there is a theoretical potential for reducing toxicity by separating radiotherapy and ICI non-simultaneously, there is no definite conclusion regarding the optimal interval time between them. Data on combination therapy for ESCC are limited, and we can draw upon our experience treating lung cancer to inform our decisions. Notably, the PACIFIC study revealed marginally improved outcomes for patients who initiated Durvalumab within two weeks after radiotherapy (126). In the real-world PACIFIC- R study, a more favorable prognosis was observed for patients who commenced Durvalumab treatment within 42 days compared to those who delayed Durvalumab initiation (127). Nevertheless, a meta-analysis also demonstrated that the interval between radiotherapy and Durvalumab frequently exceeded 42 days in practical use and did not affect 12-month PFS/OS (128). Therefore, in ongoing clinical trials investigating consolidation therapy for ESCC, the interval between the completion of radiotherapy and the initiation of ICI generally extends to 6 weeks (74, 78). The interval between esophagectomy and adjuvant immunotherapy may be longer. Perioperative immunophenotype analysis revealed that cytotoxic did not begin to grow until 6 weeks after surgery (129). The subgroup analysis of CheckMate 577 study demonstrated a superior survival benefit for commencing Nivolumab beyond 10 weeks after surgery, as opposed to initiating treatment within 10 weeks, which could be attributed to the extended period of time needed for the immune system to recuperate after neoadjuvant chemoradiotherapy and radical surgery (42). Real-world study of metastatic/recurrent ESCC found that patients treated with radiation within 90 days before and after immunotherapy exhibited extended median PFS and OS than those treated with radiation beyond 90 days (124). Clinical trials for metastatic ESCC necessitate an interval of no more than 8 weeks between conventional radiotherapy and ICI (79, 81), and one week between SBRT and ICI (91–93). A multicenter retrospective study in Italy discovered that an interval of less than 7 days between SBRT and ICIs resulted in a longer OS with slightly higher but manageable toxicity (130, 131). When utilizing stereotactic radiosurgery (SRS) to address brain metastases, concurrent ICI therapy within four weeks was found to provide the most optimal benefit (132). The most effective schedule for incorporating ICI with radiotherapy remains unknown and may vary across different multimodal treatments.

The sequencing of radio-immunotherapy combinations is also a critical determinant to their efficacy. In mouse models, investigations have demonstrated that concurrent application of PD-1 inhibitors with or soon after local radiotherapy has the potential to augment the expansion of multifunctional intratumoral CD8+ T cells and reduce peripheral CD8+ T cell death, leading to a more favorable systemic antitumor response and abscopal effect (133, 134). Patients who received ICI after or concurrently with SBRT/SRS also showed better outcomes than those who received it previously (135–137), possibly related to radiotherapy-induced neoantigen release and increased PD-L1 expression. However, certain clinical studies have found no statistical difference between simultaneous and sequential administration of SBRT and Ipilimumab (138). Ongoing clinical trials are specifically comparing the effects of different sequences (139, 140).

It remains unclear whether the exact sequence of combinations or the interval between them has a greater impact on efficacy. While most studies tend to administer ICI simultaneously with or sequentially after radiotherapy, there have been reports that anti-PD-1 administered after esophageal radiotherapy is more susceptible to perforation, necessitating careful monitoring (141). Particularly for patients with ulcerated, giant, thin-walled or large-vessel invaded esophageal cancer, induction immunotherapy can be considered first to alleviate symptoms and improve nutritional status, thus reducing the irradiation volume and associated side effects, and better protecting surrounding normal tissues.

#### 4.2.2 Dosage and fractionation regimen of radiotherapy

The optimal irradiation dose for esophageal cancer has always been controversial, with higher doses being administered in regions where ESCC is more prevalent than EAC. However, as systemic therapy has intensified in recent years, several large-scale phase III studies have compared the efficacy and safety of a 50GY radiation dose with higher doses in ESCC (13, 14, 142, 143). The current preference is for 50GY as the recommended dose of radical radiotherapy for ESCC (19). We have pondered whether the radiation dose range of 50-60GY may be too high in combination with intensified systemic therapy, and whether a lower dose would be more appropriate. To explore this question, the KEYNOTE-975 study was designed to compare the effectiveness of two radiation dose groups, 50GY and 60GY, in combination with Pembrolizumab (65). In the field of neoadjuvant therapy, the guidelines recommend a radiotherapy dose of 41.4-50.4GY (19). Higher doses do not improve the efficacy and survival (144, 145), and most of the current clinical trials employ the CROSS regimen of 41.4GY/23F. The neoadjuvant radiotherapy in the SCALE study used a short course of 30GY/12F, achieving a promising pCR rate of 55% (36). In NICE-RT study, the neoadjuvant radiotherapy regimen consisted of conventional fractionated irradiation (41.4GY/23F) for the primary lesion and adjacent lymph nodes, plus low-dose irradiation (0.5GY*4) for abscopal lymph nodes (146). Regarding metastatic ESCC, radiotherapy for the primary site mostly involves definitive or neoadjuvant radiotherapy regimens. Regimens for targeting metastatic sites are more varied, including attempts at low-dose radiotherapy (94), combination radiotherapy at different doses (147), as well as SBRT/SRS. Integration of high- and low-dose radiation (20-70GY total 3-12.5GY/F; 1-10GY total 0.5-2GY/F) has demonstrated the potential to reverse immune resistance in metastatic patients who have progressed after immunotherapy treatment (147). The ongoing PALEO study used Durvarlumab plus hypo fractionated radiotherapy (30GY/10F) in week 1-2 for primary esophageal lesions and SBRT (24GY/3F) in week 7 for a single metastasis lesion (87).

#### 4.2.3 Target volume of radiotherapy

There has long been a contentious debate surrounding the selection of involved-field irradiation versus elective nodal irradiation for delineating radiotherapy targets in esophageal cancer. Given the dependence of immunotherapy on lymphocytes, especially T-lymphocytes, and the negative impact of lymphopenia on prognosis (148–150), the prevailing tendency is to opt for involved field irradiation to preserve lymphocyte function and minimize lymph node irradiation, while minimizing side effects. Fewer studies have explored prophylactic lymph node irradiation (69).

Chang YJ has advocated for a multi-site irradiation strategy over single-site irradiation to overcome the tumor-associated antigen heterogeneity, thus improving the efficacy of radiotherapy combined with ICI (151). Several ongoing clinical studies of combined radiotherapy with ICI are utilizing multi-technique and multi-target irradiation schemes (81, 87, 93, 94, 146). The radiotherapy-induced immune response varies depending on irradiation site, highlighting the significance of selecting appropriate metastatic sites for irradiation in radio-immunotherapy. It has been observed that SBRT to parenchymal (brain, liver, lung) rather than nonparenchymal (bone) metastases is more likely to trigger systemic immune activation, and is associated with improved prognosis, possibly due to different inherent microenvironment of different metastatic organs (152–154). The optimal irradiation site remains uncertain. A phase I trial investigating the combination of SBRT and Ipilimumab demonstrated that liver metastasis irradiation was associated with a more favorable T-cell activation compared to lung metastasis irradiation (155). Moreover, other studies have shown that irradiation to brain metastases may result in the best synergism effect (153).

#### 4.2.4 Thoughts on de-radiotherapy

With the evolution of anti-tumor drugs, there has been a growing interest in investigating the potential of drug therapy to replace radiotherapy or surgery. However, previous clinical trials have shown that the pCR rate of neoadjuvant immunochemotherapy typically ranges between 25-45% (156–164), which was lower compared to conventional chemoradiotherapy (165). The pCR rate of neoadjuvant chemoradiotherapy combined with ICI is mostly above 45%, with the highest rate of 55.6% observed in the PALACE-1 study (35). A meta-analysis indicated that neoadjuvant immunochemoradiotherapy led to a higher pCR rate compared to immunochemotherapy for ESCC, and increasing the cycles of ICI did not appear to improve the pCR rate (166). Given that the absence of preoperative radiotherapy may result in a lower pCR and subsequently impact the prognosis, the role of radiotherapy in neoadjuvant therapy is still considered essential.

Several ongoing clinical trials are comparing neoadjuvant immunochemotherapy with neoadjuvant chemoradiotherapy, such as KEYSTONE-002 study (NCT04807673) (167), NICE2 study (NCT05043688) (168) and REVO study (NCT05007145) (169). The NICE 2 study was divided into three groups to compare the efficacy and safety of three neoadjuvant treatment regimens: immunotherapy combined with chemotherapy, immunotherapy combined with chemoradiotherapy and conventional chemoradiotherapy (168). Another study (NCT05624099) compared Camrelizumab plus chemoradiotherapy versus Camrelizumab plus chemotherapy (170). The VESTIGE trial is evaluating the efficacy of adjuvant chemotherapy with adjuvant Nivolumab plus Ipilimumab in esophageal cancer with high risk of recurrence after neoadjuvant chemotherapy and surgery. Compared with CheckMate 577 trial, this trial excluded patients who had received radiotherapy, and may provide insights into the role of radiotherapy and its synergistic effect with immunotherapy (171). Additionally, the clinical trials NCT05637268 and NCT04741490 are recruiting patients with R0-resected ESCC and comparing the outcomes of adjuvant therapy using either immunotherapy combined with chemotherapy or immunotherapy combined with radiotherapy (59, 172). The results of these studies will provide further evidence to determine the optimal combination strategy for neoadjuvant or adjuvant treatment of ESCC and clarify the role of radiotherapy in this setting.

### 4.3 Thoughts on de-surgery

For resectable or potentially resectable locally advanced ESCC, the current standard of care is neoadjuvant chemoradiotherapy followed by surgery. However, due to concerns about surgical complications and reduced postoperative quality of life, not all patients ultimately undergo surgery, especially those who achieve clinical complete remission (cCR) after neoadjuvant chemoradiotherapy. Based on data from several large clinical studies, approximately half of ESCC patients can achieve pCR after neoadjuvant chemoradiotherapy (8, 10), and this high pCR rate provides a theoretical basis for exploring regular surveillance without surgery. Can esophageal cancer learn from the “watch and wait” strategy of rectal cancer in complete remission after neoadjuvant chemoradiotherapy, and use esophagectomy as a salvage method for local recurrence? Multiple retrospective studies have demonstrated that among good responders after neoadjuvant therapy, active surveillance of patients who refused esophagectomy or were ultimately deemed unsuitable for surgery had comparable survival rates to those who underwent surgery (173–178). In the ongoing prospective SANO trial and ESOSTRATE trial, patients with a clinical response assessment of cCR after standard neoadjuvant chemoradiotherapy will be randomly assigned to either the active surveillance group or the immediate surgery group, comparing survival and quality of life for both groups (179–181). Neoadjuvant chemoradiotherapy combined with immunotherapy has achieved a pCR rate exceeding 50% potentially enabling more responders to bypass surgery, thereby retaining their esophageal function and improving survival with a higher quality of life. The ongoing Phase II WATCHER trial enrolled patients who achieved cCR after neoadjuvant chemoradiotherapy plus Camrelizumab, comparing survival differences between the surgery group and watch-and-wait group, and assessing the effect of immunotherapy maintenance (182).

Until the results of clinical trials are published, the standard treatment for locally advanced ESCC with a clinically complete response remains primarily surgical. The decision to withdraw surgery must be made with caution, with adequate communication with the patient, enhanced surveillance, and prompt remediation if necessary. Additionally, it is essential to acknowledge that accurate and safe response assessment after neoadjuvant therapy is crucial for clinical decision-making, and a single test is insufficient. A combination of CT/MRI findings, endoscopic ultrasonography, bite-on-bite biopsies, fine-needle aspiration of suspicious lymph nodes, and serial PET-CT for dynamic monitoring of distant metastases is necessary (183–185). Even with the combined application of CT, PET-CT and endoscopic biopsy, half of the patients eligible for cCR for surgery still have pathological residual tumors, especially in the lymph nodes, which are more likely to be underestimated (186). Under current conditions, surgery can provide accurate pathologic staging information and facilitate clinicians to assess risk stratification and target adjuvant therapy.

### 4.4 Treatment-related adverse events

The incidence of high-grade AEs in combination therapy ranges from 20% to 50%, with lymphocytopenia being the most common, followed by esophagitis, anastomotic leak, and esophageal fistula (131, 166, 187–191). According to data from the Food and Drug Administration of the United States, grade 3-4 pneumonitis occurred in 1.1%, 1.9%, and 1.2% of patients who did not receive radiotherapy, received ICI within 90 days of radiotherapy, and received ICI more than 90 days after radiotherapy, respectively. Although radiotherapy combined with ICI may increase the incidence of severe radiation pneumonitis, the absolute percentage increase is so small that the administration of ICI within 90 days after radiotherapy appears to be safe (192). No statistic difference was observed in the risk of major complications, such as pulmonary complications, anastomotic leakage, and other complications, as well as death or readmission, among patients who received neoadjuvant chemoradiotherapy combined with or without immunotherapy (193). However, it was reported that 61% of patients with immune-related AEs history developed grade 2 or higher radiation pneumonitis after radiotherapy, and 83% of patients with prior ICI pneumonitis developed grade 2 or higher radiation pneumonitis (194). A retrospective study also found that radiotherapy combined with anti-PD-1 increased the incidence of esophageal perforation (18% vs. 3.1% p=0.002) (141). Although most clinical trials have deemed the safety of combination therapy to be acceptable, the populations enrolled in these trials typically have lower tumor burdens and better performance status, which may not reflect the daily clinical practice where AEs may be more common. Despite most clinical trials deeming the safety of combination therapy acceptable, the enrolled populations typically have lower tumor burdens and better performance status, which may not accurately reflect the occurrence of AEs in daily clinical practice.

## 5 Conclusions

The combination of immunotherapy and radiotherapy has emerged as a promising treatment approach in esophageal cancer that warrants further investigation and optimization. For locally advanced resectable ESCC, adding immunotherapy to standard care of the neoadjuvant chemoradiotherapy followed by surgery has not significantly increased the pCR rate but has increased toxicity to some extent. Evidence for neoadjuvant immunotherapy is limited to small-scale single-arm phase I/II trials and is not yet suitable for widespread application. Further research is needed to explore the optimal combination strategy for neoadjuvant treatment. In terms of postoperative adjuvant immunotherapy, the ChckMate577 trial has provided conclusive evidence, yet further exploration is necessary in terms of target populations, dosing regimens, and predictive biomarkers. For locally advanced unresectable ESCC, the addition of immunotherapy to definitive chemoradiotherapy during the concurrent or consolidation phase holds promise for improved long-term survival. For metastatic ESCC, aggressive multidisciplinary treatment combing radiotherapy to immunochemotherapy is important for symptom improvement and survival prolongation, especially for ESCC with oligometastasis.

Preliminary results of radio-immunotherapy for ESCC are promising, with a succession of large-scale studies currently underway. Issues such as irradiation dosage, fractionation regimen, irradiation site and technique of radiotherapy, the timing, sequence and duration of combination therapy, and the selection of immunotherapeutic agents will all affect treatment outcomes. We need to keep on exploring the anti-tumor mechanism and predictive markers of radio-immunotherapy. With the development of multi-omics and pharmaceutical technology, precision screening, accurate assessment and multidisciplinary treatment will certainly improve significantly, and the day of preserving organ function with high quality of life and long-term survival for esophageal cancer patients will not be far away.

## Author contributions

MJ and HL contributed to the conception and design of the review. MJ, YH, GL and CC contributed to the data acquisition, data analysis and interpretation, original draft preparation. MJ, CC and HL contributed to review and revise the manuscript. All authors contributed to the article and approved the submitted version.
